# Evaluating the potential synergistic benefit of a realignment brace on patients receiving exercise therapy for patellofemoral pain syndrome: a randomized clinical trial

**DOI:** 10.1007/s00402-016-2464-2

**Published:** 2016-05-05

**Authors:** Wolf Petersen, Andree Ellermann, Ingo Volker Rembitzki, Sven Scheffler, Mirco Herbort, Gert Peter Brüggemann, Raymond Best, Thore Zantop, Christian Liebau

**Affiliations:** Klinik für Orthopädie und Unfallchirurgie, Martin Luther Krankenhaus Berlin, Grunewald, Caspar Theyß Strasse 27-31, 14193 Berlin, Germany; Arcus Sportklinik, Pforzheim, Germany; Otto Bock, Duderstadt, Germany; Asklepios, Harzkliniken GmbH, Fritz-König-Stift, Bad Harzburg, Germany; Sporthopaedicum Berlin, Berlin, Germany; Klinik für Unfall-, Hand-, und Wiederherstellungschirurgie, Universitätsklinikum Münster, Münster, Germany; Deutsche Sporthochschule Köln, Institut für Biomechanik, Cologne, Germany; Sportklinik Stuttgart, Stuttgart, Germany; Sporthopaedicum, Straubing, Germany

**Keywords:** Patellar maltracking, Dynamic valgus, Anterior knee pain, Functional malalignment, Chondromalacia patellae, Patellar orthosis, Patellar tape

## Abstract

**Background:**

It has been previously shown that exercise programs for patellofemoral pain syndrome (PFPS) can be supported by medially directed taping. Evidence supporting the use of patellar braces is limited because previous studies have been low quality. The aim of this study is to compare the outcomes of patients with PFPS after treatment with a medially directed patellar realignment brace and supervised exercise.

**Methods:**

In a prospective randomized multicenter trial, 156 patients with PFPS were included and randomly assigned to 6 weeks of supervised physiotherapy in combination with the patellar realignment brace, or supervised physiotherapy alone. Outcome measures were the Knee Injury and Osteoarthritis Outcome Score (KOOS) subscales, numeric analog pain scores, and the Kujala score at baseline, 6 weeks, 3 months, and 1 year after the start of therapy. The patient’s self-reported perception of recovery was also assessed at these points.

**Results:**

Both treatment groups showed a significant improvement in all outcome measures over the study period. After 6 and 12 weeks of therapy, patients in the brace group had significantly higher KOOS sub-scale scores, a higher mean Kujala score, and less pain while climbing stairs or playing sports. After 54 weeks a group difference could be only detected for the KOOS ADL sub-scale.

**Conclusion:**

The use of a medially directed realignment brace leads to better outcomes in patients with PFPS than exercise alone after 6 and 12 weeks of treatment. After 1 year of follow-up this positive effect diminished.

## Introduction

Patellofemoral pain syndrome (PFPS) is a frequent cause of anterior knee pain [5, 11]. Several studies have shown that PFPS mainly affects patients who do not have significant cartilage damage [7, 18, 25]. Despite the lack of structural pathology, PFPS forces many athletes to limit their sport activities [5].

There is no consensus concerning the etiology of PFPS [25]. Several studies, however, suggest that patella maltracking probably plays a role in the pathogenesis of PFPS [17, 24, 36]. There is evidence in the literature that the cause of patellar maltracking may be structural in nature [25]. A systematic review has shown that altered frontal plane biomechanics is an important risk factor associated with PFPS [35]. Dynamic valgus forces due to the internal rotation of the femur rather than static valgus forces (torsion) may influence patellar tracking and lead to the lateralization and tilt of the patella [24]. Weakness of the hip external rotators and abductors (gluteus medius and minimus) is considered a key factor predisposing patients to the development of PFPS [2, 6, 9]. Other muscular imbalances involving the quadriceps and hamstrings have been described, but are likely to be secondary in nature [13].

Based on this pathogenesis, exercises can be a causal therapeutic approach for PFPS [28, 32]. Several meta-analyses have shown the positive effects of exercise on pain reduction in patients with PFPS [21, 28, 32].

Another therapeutic approach for PFPS is to correct patellar maltracking with the help of tape or patellar braces [8, 34]. Patellar braces are non-adhesive devices that, like taping, apply a medially directed force that may counteract lateral patellar maltracking [29, 34]. It has been shown that bracing and taping provide coronal-plane and torsional control of the patella during the eccentric contraction of the quadriceps in both PFPS patients and healthy subjects [4, 27, 37]. Other authors showed that there was a significantly higher level of neuromotor and proprioceptive function with the application of a patellar brace [11, 31].

Evidence of the clinical effect of patellar braces should be regarded as limited due to the low quality of previously published studies [8, 34]. A meta-analysis published by Warden et al. [34] found that only one of three included studies reported a statistically significant impact of a medially directed patella brace.

**Fig. 1:**
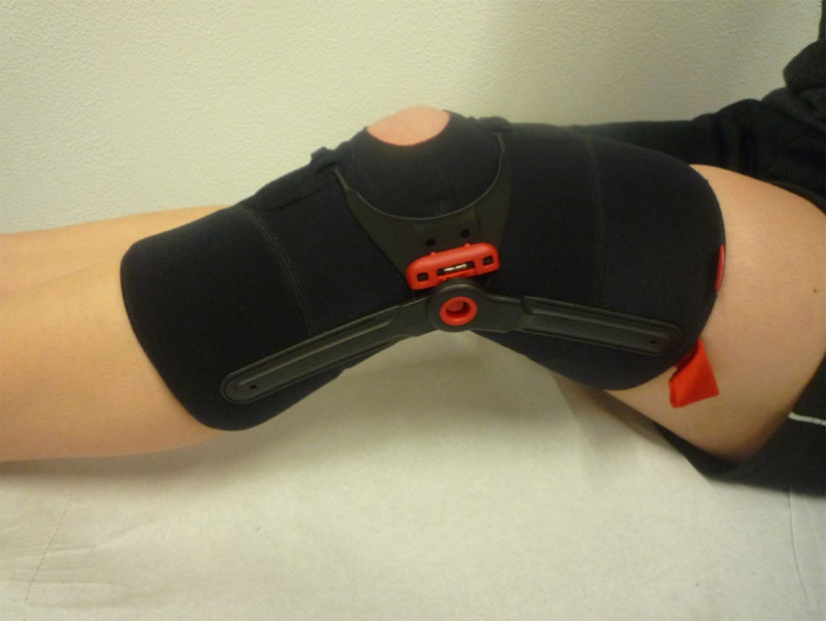
The patellar realignment brace (Patella Pro, Otto Bock, Duderstadt, Germany). The sleeve of the brace applies a medially directed force on the patella

It was therefore the aim of the present study to perform a prospective randomized trial to evaluate the effect of a new realignment brace on patients with PFPS who were treated with physiotherapy [26]. The realignment brace (Patella Pro, Otto Bock, Duderstadt, Germany) is a knee brace that applies a medially directed force to counteract lateral patella maltracking and tilt (Fig. 1). The tracking system of this orthosis was designed to control patella tracking only within 0°–30° of flexion. In this range patella tracking is not guided by the trochlea [26]. The design of this brace is advantageous, as the pressure applied by the tracking system decreases with increasing flexion angle [10]. The biomechanical effect of this brace was demonstrated in two previously published studies [4, 10].

The hypothesis of the present study is that there is a synergistic effect of the use of a realignment brace and physiotherapy in patients with PFPS.

## Methods/design

### Study design

This study is a randomized multicenter clinical trial examining the short-term effectiveness of a patellar brace (Patella Pro, Otto Bock, Duderstadt, Germany) in combination with exercise on short and longterm PFPS outcomes compared with exercise alone.

The study protocol was approved by the medical ethics committee of Charite University Hospital. The study protocol was registered with the *Deutsches Register Klinischer Studien* (“German Clinical Trials Register”) as DRKS-ID number DRKS00003291 and published [26]. All research was conducted in accordance with the 1964 Helsinki Declaration. The protocol of a previous study that evaluated the effects of exercise on patients with PFPS served as the model for the study design of the present study [33].

Patients were recruited from the following study centers: (1) Klinik für Orthopädie und Unfallchirurgie, Martin Luther Krankenhaus, Berlin Grunewald, Germany, (2) Arcus Sportklinik, Pforzheim, Germany, (3) Asklepios, Harzkliniken GmbH, Fritz-König-Stift, Bad Harzburg, Germany, (4) Orthopädische Gemeinschaftspraxis, Berlin, Germany, (5) Klinik für Unfall-, Hand-, und Wiederherstellungschirurgie, Universitätsklinikum Münster, Germany, and (6) Orthopädische Klinik, Rosenheim, Germany [26].

The recruitment period took place from April 2012 to October 2014. Adult patients aged 18–50 years with PFPS symptoms for longer than 2 months but not longer than 2 years were eligible to participate [26].

Inclusion criteria consisted of a patient age between 18 and 50 years and the presence of three of the following symptoms lasting longer than 2 months but not longer than 2 years: anterior knee pain when running, climbing stairs, cycling, sitting with a bent knee, or performing squats [26].

Exclusion criteria consisted of the following: Kellgren-Lawrence grade 3 to grade 4 osteoarthritis [22], local grade 3 to grade 4 cartilage damage as noted on magnetic resonance imaging and measured using the Gluckert grading system [20], subluxation of the patella, a history of a previous knee injury (such as to the cruciate ligaments), tendinosis of the patellar tendon, a history or active diagnosis of Osgood–Schlatter disease, osteochondritis dissecans, a varus knee with an intercondylar distance greater than 2 fingerbreadths, and a valgus knee an intermalleolar distance greater than 3 fingerbreadths [26].

Patients who qualified as study participants on the basis of the inclusion and exclusion criteria were informed about the study design. Informed consent was obtained from all individual participants included in the study.

Our sample size calculation was based on a former intervention study by Clark et al. [14]. In this study the difference in recovery rates between the intervention and control groups was 22 %. This difference was statistically significant (power 0.8, alpha 0.05). With a potential dropout rate of about 15 %, approximately 156 patients must be enrolled in this study to achieve a power of 0.80 and an alpha of 0.05.

After patients were recruited and informed consent was obtained, all patients were randomized into two treatment groups. In group 1 (brace group) all patients received a patellar brace (Patella Pro) (Fig. 1). With this brace, a medially directed force can be applied to the patella by a tracking system. The brace was customized for the patient by the study physician. Patients were instructed to wear the brace over a minimum period of 6 weeks for at least 6 h a day. In group 2 (non-brace group) no brace was applied and patients were instructed to not utilize a brace over the 6-week study period [26].

In both groups, patients entered a supervised exercise and structured home exercise program (Patella Move program). Patients were instructed to perform the home exercises daily for 15 min for a period of 6 weeks. For supervised exercises, all study participants received a prescription of about 12 sessions of *Krankengymnastik am Gerät*. The duration of one session is 60 min [26]. The duration of the supervised exercise program was 6 weeks (12 units).

During the course of the study, the application of ice and topical agents, and the use of oral analgesics (e.g., non-steroidal anti-inflammatory drugs or paracetamol) was permitted and recorded.

### Outcome measures

The primary outcome measures for this study were subjective assessments of recovery using a seven-point Likert scale [33] administered at 6, 12, and 54 weeks following the start of therapy. This parameter was used for the sample size calculation. Secondary outcome measures included the modified functional Kujala score without the muscular atrophy and flexion parameters, the German version of the Knee Injury and Osteoarthritis Outcome Score (KOOS) [23], pain at rest and with walking, stair climbing, sitting and sports activity, reported on a numerical scale (0–100) [15], and a review of additional interventions. All these measurements were evaluated via questionnaire prior to any intervention, and 6, 12, and 54 weeks after the start of therapy.

All patients were asked after 6 weeks of therapy if they had adhered to the treatment protocol (6 h per day of the patella brace for 6 weeks, 15 min of home exercise daily, and 12 sessions of physiotherapy) [26].

### Statistics

All statistical analysis was performed by a contract statistician (Medi Stat, Kiel, Germany). To evaluate the additional effects of a patellar brace on supervised exercise and home exercise in patients with PFPS, between-group differences in clinical outcomes were analyzed. The Kolmogorov–Smirnov-test was used to test the groups for a normal distribution. The Mann–Whitney *U* test was used as a non-parametric test. The Chi square test was used for a parametric distribution.

## Results

### Recruitment, inclusion and follow-up

**Fig. 2:**
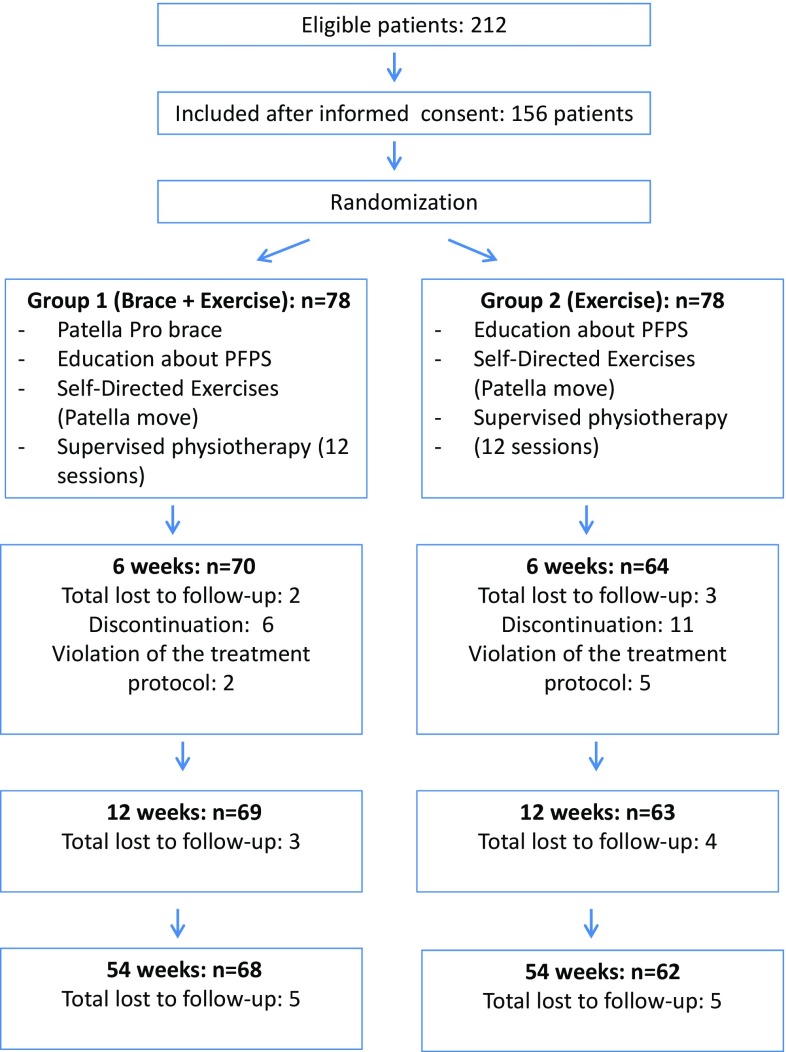
Flow chart of the study design

Figure 2 shows a flow chart illustrating patient recruitment, inclusion, and follow-up. A total of 156 patients were eligible for inclusion in the present study.

Six patients from the brace group and eleven from the non-brace group discontinued their participation in the study. In all cases, the reason for patient discontinuation was a lack of motivation. Seven further patients admitted to partial violations in the protocol (less than 50 % brace use, home exercises or physiotherapy than recommended). In the brace group, two, three and five patients were lost to follow-up after 6, 12 and 54 weeks respectively. In the non-brace group, three, four and five patients were lost to follow-up after 6, 12 and 54 weeks respectively (Fig. 2).

There was no statistical difference in gender distribution between the two study groups (Chi square test, *p* = 0.079), and no statistical differences between age and body mass index (BMI, Kolmogorov–Smirnov-test, *p* < 0.05) (Table 1).

**Table 1:** Demographic characteristics of the brace and control groups

	Mean
Brace group	
Age (years)	28.0 (±9.4)
BMI (kg/m^2^)	23.0 (±1.5)
Females (%)	65.8
Non-brace group	
Age (years)	28.0 (±8.1)
BMI (kg/m^2^)	23.0 (±1.3)
Females (%)	78.9

### Pain

**Fig. 3:**
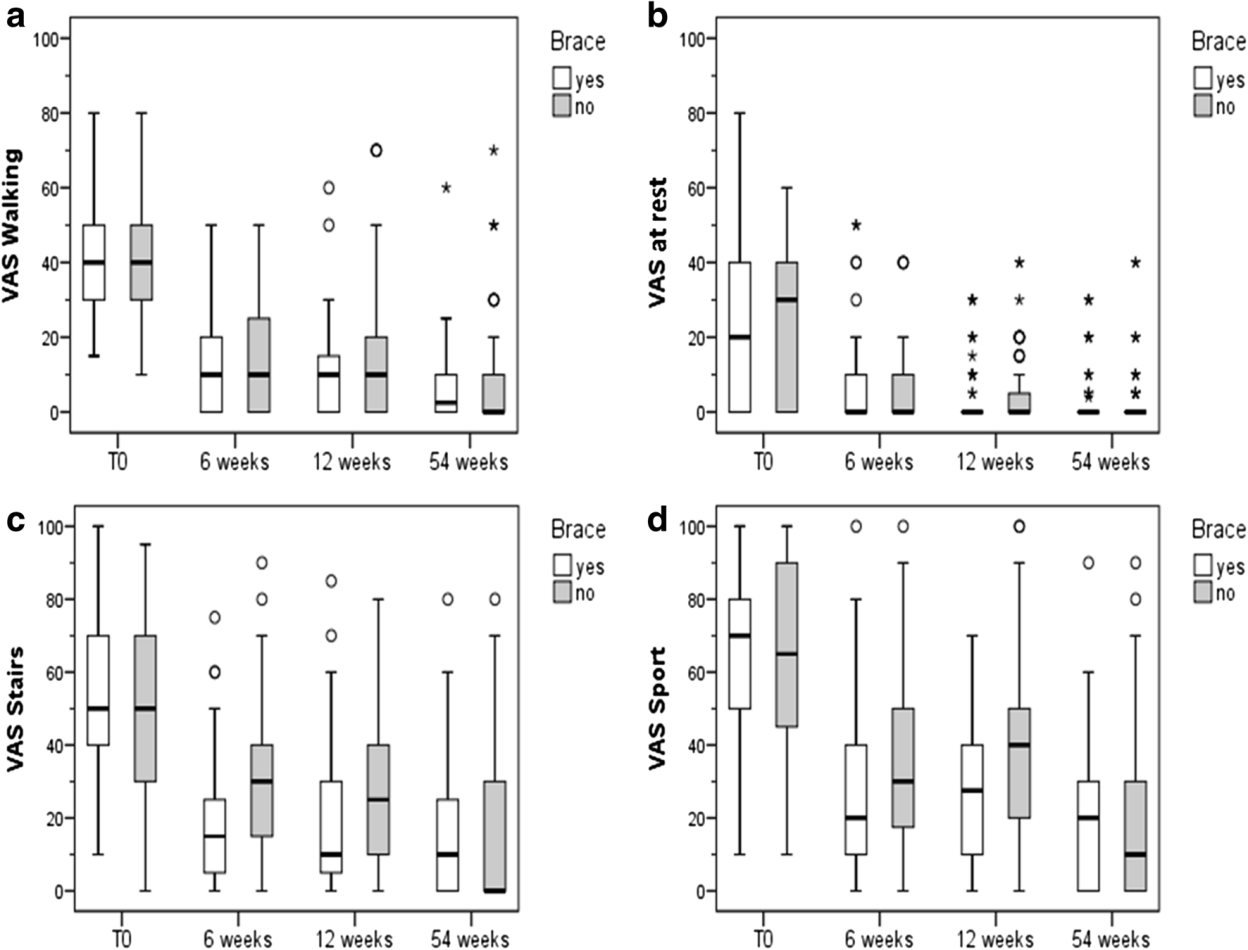
Pain assessed on a numerical analog scale. For all parameters a non-parametric distribution was found (Kolmogorov–Smirnov-test, *p* < 0.05). **a** In both groups pain with walking improved over time (Friedman-test, *p* < 0.001). No between-groups differences could be detected (*U* test, *p* ≥ 0.05). **b** Pain at rest improved in both groups (Friedman-test, *p* < 0.001). No between-groups differences could be detected at any time point (*U* test, *p* ≥ 0.05). **c** In both groups pain during stair climbing improved significantly over time (Friedman-test, *p* < 0.001). Significant between-group differences could be detected after 6 (*U* test, *p* = 0.002) and 12 (*p* = 0.003) weeks of intervention. **d** Pain during sports improved in both groups over time (Friedman-test, *p* < 0.001). Significant between-group differences could be detected after 12 weeks of intervention (*U* test, *p* = 0.003)

Pain (numerical analog scale: NAS) was assessed at rest and while walking, climbing stairs, and playing sports (Fig. 3). In both treatment groups there was a significant improvement in pain with all four activities over time.

No significant group differences could be detected during walking and at rest after 6, 12, and 54 weeks. Absolute and percent changes in pain at rest and while walking also did not differ significantly between the brace and non-brace groups.

However, significant lower limb pain was assessed while climbing stairs or playing sports for the brace group compared to the non-brace group after 6 and 12 weeks. The absolute and percent decrease in reported pain also differed significantly between the brace and non-brace groups in week 6 and 12. After 54 weeks, no significant differences between both treatment groups were noted.

### KOOS subscales

**Fig. 4:**
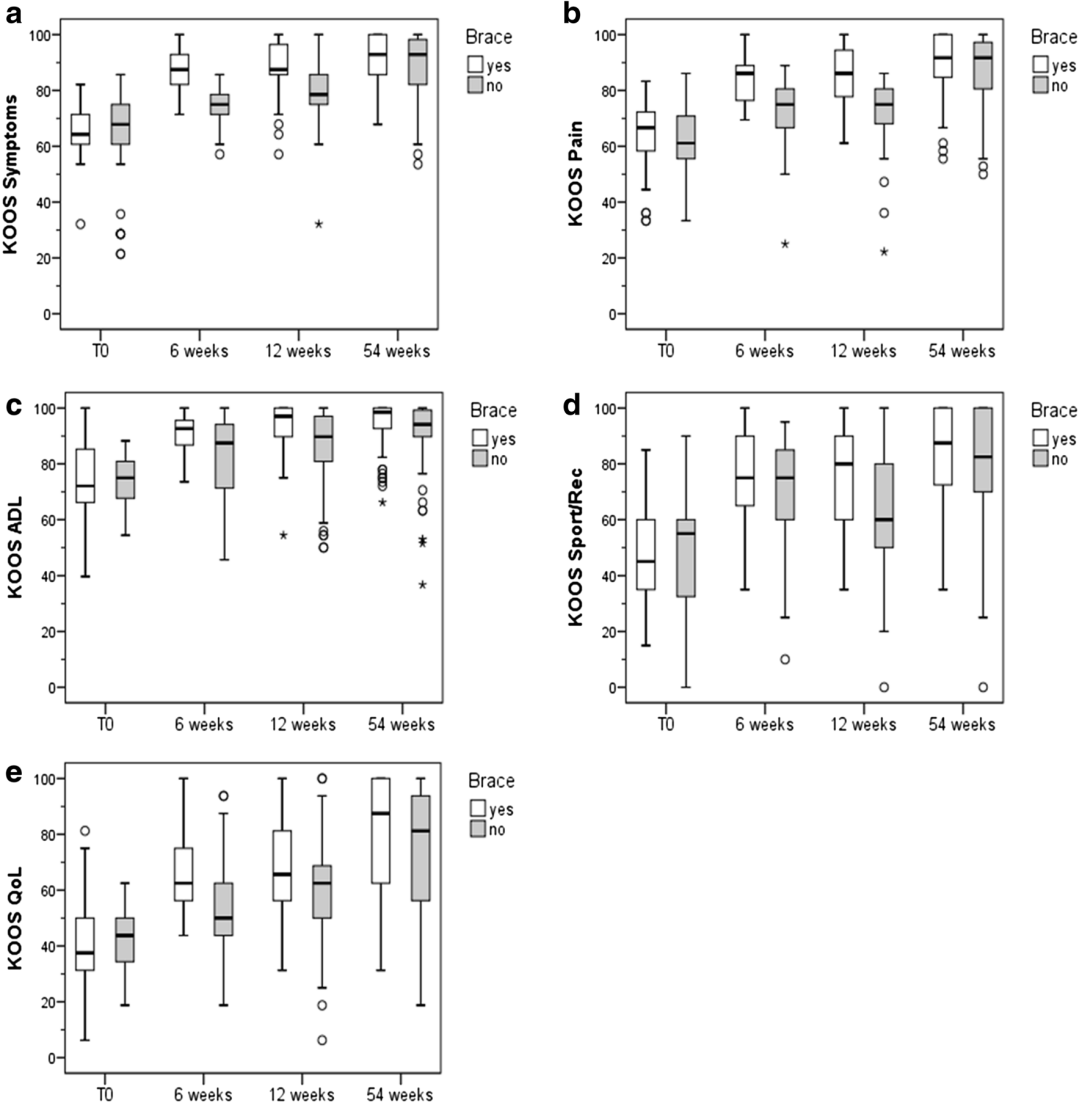
Results of the five KOOS subscales. Non-parametric tests were used for all five KOOS subscales (Kolmogorov–Smirnov-test, *p* < 0.05). **a** KOOS symptoms: In both groups the KOOS symptoms sub-score increased significantly (Friedman-test, *p* < 0.001). Significant between-group differences could be detected after 6 (*U* test, *p* < 0.001) and 12 (*p* < 0.001) weeks of intervention. **b** In both groups the average KOOS pain values increased from T0 to all three follow-up examinations (Friedman-test, *p* < 0.001). Significant between-group differences could be detected after 6 (*U* test, *p* < 0.001) and 12 weeks (*p* < 0.001) of intervention. **c** In both groups, the KOOS ADL sub-score increased over all time points (Friedman-test, *p* < 0.001). Significant between-group differences could be detected after 6 (*U* test, *p* = 0.002), 12 (*p* < 0.001) and 54 weeks (*p* = 0.034) of intervention. **d** In both groups, the KOOS sports/rec sub-score increased over all follow-up time points (Friedman-test, *p* < 0.001). Significant between-group differences could be detected after 6 (*U* test, *p* = 0.038) and 12 (*p* = 0.001) weeks of intervention. **e** In both groups, the KOOS QoL sub-score increased over all follow-up time points (Friedman-test, *p* < 0.001). Significant between-group differences could be detected after 6 (*U* test, *p* = 0.001) and 12 (*p* = 0.011) weeks of intervention

Figure 4 shows the survey measurements of the five KOOS subscales. All five KOOS subscales increased significantly in both treatment groups over all three follow-up time points.

Significantly higher scores in the brace group could be detected for the pain, symptoms, activities of daily living (ADL) and quality of life (QoL) sub-scores at the 6- and 12-week time points. For the sports/recreational activities (Sport/Rec) sub-score, a significantly higher score could only be found after 12 weeks. After 54 weeks, significant group differences could only be found in the ADL sub-score.

### Kujala score

**Fig. 5:**
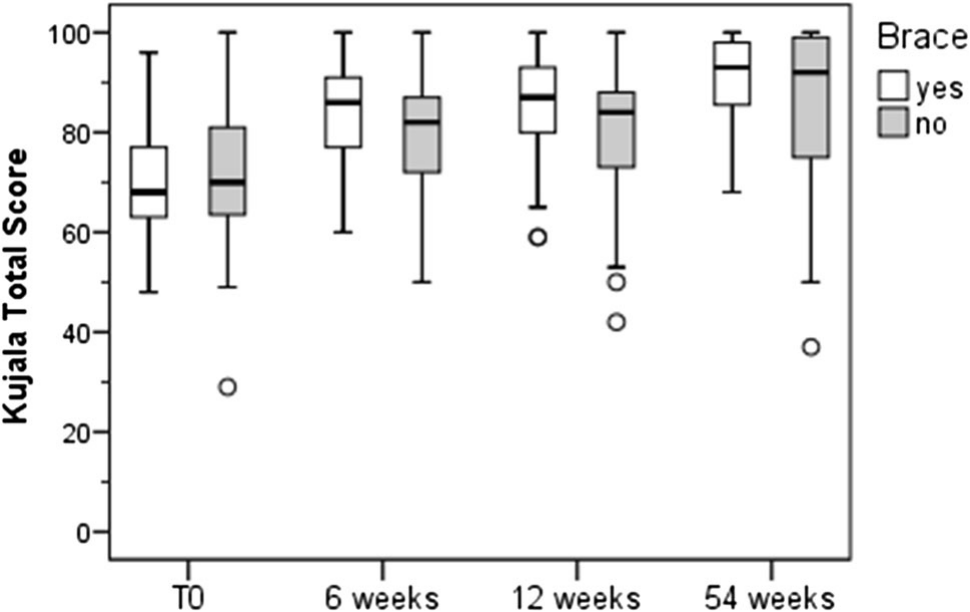
Kujala score. In both groups the Kujala-score increased over all three follow-up time points (Friedman-test, *p* < 0.001). Significant between-group differences were detected after 6 (*U* test, *p* = 0.029) and 12 (*p* = 0.037) weeks of intervention

Figure 5 shows the results of the Kujala score. There was a significant improvement in the mean Kujala score with and without the brace over time. Between-group differences could be detected at 6 and 12 weeks. A significantly higher mean Kujala score compared with pre-therapy measurements was found for the brace group compared to controls at 6 and 12 weeks.

### Recovery

In both groups, a higher proportion of patients reported recovery after 6, 12 and 54 weeks. However, no significant between-group differences could be detected.

### Additional interventions

There was no significant difference between the intervention group and the control group in the number of patients who used oral or topical NSAIDs as additional interventions (Table 2).

**Table 2:** Additional interventions required by members of the brace and non-brace groups

	Additional interventions	Brace group (number of patients)	Non-brace group (number of patients)
6 weeks	NSAIDs	12	14
	Topical agents	2	3
12 weeks	NSAIDs	8	11
	Topical agents	2	2
54 weeks	NSAIDs	3	3
	Topical agents	1	1

## Discussion

The results of the present study confirm our hypothesis that there is a synergistic effect of physiotherapy and a realignment brace during the treatment phase of patients with PFPS. In both treatment groups the results of the KOOS subscales, functional Kujala score and pain ratings during activities such as climbing stairs or sports improved compared to baseline. At 6- and 12-week follow-up, these scores were significantly better in braced patients than in non-braced patients. After 1 year there was no significant difference in the overall outcomes of the two treatment groups.

Physiotherapy is an established treatment modality for patients with PFPS because exercise is the causal treatment to correct dynamic valgus stress in patients with PFPS. The improvements noted in the clinical scores of both treatment groups are in accordance with previous studies [14, 33]. A Cochrane review has found evidence that exercise therapy for PFPS is beneficial for pain reduction, functional improvement, and long-term recovery [32]. However, the best form of exercise therapy for patients with PFPS is still unknown [32]. In the present study, both treatment groups performed a complex supervised exercise program to improve the strength, coordination, endurance, and flexibility of the lower extremity, including the hip muscles. In addition, both groups were instructed to perform home exercises on their own in a structured program (Patella Move). This study therefore does not permit us to make any conclusions about the best form of physiotherapy.

There is less evidence from randomized trials about the effects of patellar taping and bracing [16, 30, 34]. Selfe et al. [29] could show that PFPS patients had improved coronal-plane and torsional control of the patella following the initiation of bracing and taping. However, a recent Cochrane review has found a lack of evidence supporting the use of knee orthoses for treating PFPS [30]. In a meta-analysis published by Warden et al. [34], one of the three studies utilized found a statistically significant impact of a medially directed patella brace, whereas in the other two studies, this effect was not significant. A recent randomized study showed that in general, orthoses reduce pain and improve the performance of activities of daily living [19]. A recent biomechanical study suggested that the application of a patellar brace decreased the pain of patients with PFPS while improving their walking speed and step length [1]. A synergistic effect has also been found for physiotherapy and medially directed patellar taping [3]. The simultaneous application of restraining tape and a physiotherapy exercise program achieved better outcomes than tape application alone [3].

The conflicting previously published results evaluating the effects of patellar bracing could also be the result of different brace designs. In the present study the Patellar Pro brace (Otto Bock, Duderstadt, Germany) was used for the treatment of PFPS. This brace applies a medially directed force to counteract lateral patella maltracking and tilt within a range of 0°–30° of flexion. In this range of motion patella tracking is not guided by the trochlea [26]. The advantage of this dynamic tracking system is that the pressure applied by the tracking system decreases with increasing flexion angle. The biomechanical effect of this brace was demonstrated in two previously published studies [4, 10]. Brüggemann et al. [10] showed in a cadaver a more medial patellar tracking after application of the Patellar Pro brace in contrast to an elastic bandage. Becher et al. [4] showed that the Patellar Pro brace reduces patella tilt and lateral translation in patients with lateral patella maltracking between 0° and 30° of flexion. The design of the patella brace used in the present study may be one explanation for the positive results we report here. However, we can only speculate about the importance of the biomechanical effects of the realignment brace used in the present study. Placebo, proprioceptive, or sensory skin effects may also contribute to the beneficial effects of the brace. Some authors have demonstrated improved proprioception and altered brain activity after the application of braces or tape [12, 29].

This study has also several limitations. One limitation of the present study design is that only patients with existing magnetic resonance imaging and X-rays are included. This could be a selection bias, as only those patients with a more pathologic condition would be selected [24]. Selection bias is a general limitation of randomized trials. However, the inclusion rate of eligible patients was high in this study. A second limitation could be the age range (18–50 years). This group may have additional sources for their pain complaints. However, the exclusion criteria removed some of these. A third limitation is that the study was not double-blinded. The knowledge of which patient belonged to the brace group could influence our measured outcomes. However, blinding to treatment was not possible in our study. The differences between the brace and the control group could be further influenced by differences in their compliance with treatment. Wearing the brace can motivate the patient to perform more exercises. Discontinuation of the study was more frequent in the non-brace group. Compliance with the treatment protocol was better in the brace group, although this difference was not statistically significant.

Despite these limitations, the results of this study allow us to make the conclusion that there is a synergistic effect of a patellar realignment brace and exercise for patients with PFPS, which is most important during the first 3 months after the beginning of treatment.
